# How to select a proper early warning threshold to detect infectious disease outbreaks based on the China infectious disease automated alert and response system (CIDARS)

**DOI:** 10.1186/s12889-017-4488-0

**Published:** 2017-06-12

**Authors:** Ruiping Wang, Yonggen Jiang, Engelgau Michael, Genming Zhao

**Affiliations:** 1 School of Public Health, Fudan University, NO. 130 Dong-An Road, Shanghai, 200032 China; 2 Songjiang Center for Disease Control and Prevention, Shanghai, China; 3 Center for Disease Control and Prevention, Atlanta, GA USA

**Keywords:** Moving percentile method (MPM), Proper threshold, Early-alert signal, CIDARS

## Abstract

**Background:**

China Centre for Diseases Control and Prevention (CDC) developed the China Infectious Disease Automated Alert and Response System (CIDARS) in 2005. The CIDARS was used to strengthen infectious disease surveillance and aid in the early warning of outbreak. The CIDARS has been integrated into the routine outbreak monitoring efforts of the CDC at all levels in China. Early warning threshold is crucial for outbreak detection in the CIDARS, but CDCs at all level are currently using thresholds recommended by the China CDC, and these recommended thresholds have recognized limitations. Our study therefore seeks to explore an operational method to select the proper early warning threshold according to the epidemic features of local infectious diseases.

**Methods:**

The data used in this study were extracted from the web-based Nationwide Notifiable Infectious Diseases Reporting Information System (NIDRIS), and data for infectious disease cases were organized by calendar week (1–52) and year (2009–2015) in Excel format; Px was calculated using a percentile-based moving window (moving window [5 week*5 year], x), where x represents one of 12 centiles (0.40, 0.45, 0.50….0.95). Outbreak signals for the 12 Px were calculated using the moving percentile method (MPM) based on data from the CIDARS. When the outbreak signals generated by the ‘mean + 2SD’ gold standard were in line with a Px generated outbreak signal for each week during the year of 2014, this Px was then defined as the proper threshold for the infectious disease. Finally, the performance of new selected thresholds for each infectious disease was evaluated by simulated outbreak signals based on 2015 data.

**Results:**

Six infectious diseases were selected in this study (chickenpox, mumps, hand foot and mouth diseases (HFMD), scarlet fever, influenza and rubella). Proper thresholds for chickenpox (P75), mumps (P80), influenza (P75), rubella (P45), HFMD (P75), and scarlet fever (P80) were identified. The selected proper thresholds for these 6 infectious diseases could detect almost all simulated outbreaks within a shorter time period compared to thresholds recommended by the China CDC.

**Conclusions:**

It is beneficial to select the proper early warning threshold to detect infectious disease aberrations based on characteristics and epidemic features of local diseases in the CIDARS.

## Background

Public health emergencies, especially infectious disease outbreaks have enormously affected humankind [1, 2], and infectious diseases remain the major cause of morbidity and mortality in China [3]. The Chinese Ministry of Health believes that outbreak early detection and rapid control actions are important strategies for infectious disease control and prevention [4]. In order to enhance infectious disease surveillance, the China Centre for Disease Control and Prevention (China CDC) established a web-based Nationwide Notifiable Infectious Diseases Reporting Information System (NIDRIS) in 2004 [5]. According to the Law on Prevention and Control of Infectious Disease in China, all cases of notifiable infectious disease are diagnosed by clinicians in all levels of healthcare institutions using the uniform case definition issued by the Chinese Ministry of Health, and clinicians are obligated to report all diagnosed infectious disease cases through the NIDRIS in real time. The NIDRIS is under monthly positive quality control and inspection by local CDC staffs, the unreported rate is far below 3‰ in Shanghai and 8 ‰ in China. The successful establishment of the NIDRIS has provided a cornerstone for the implementation of automated and timely detection of infectious disease aberrations [6]. In 2005, the China CDC developed the China Infectious Disease Automated-alert and Response System (CIDARS), and the CIDARS was successfully implemented and has become operational nationwide since 2008 [7]. Now the CIDARS has been integrated into the routine outbreak monitoring efforts of the CDCs at all levels (including the national level, provincial level, municipal level, and county or district level) in China [8].

The CIDARS was developed based on the NIDRIS which includes 30 infectious diseases. The 30 infectious diseases were classified into two types according to their severity, morbidity and public health importance [8, 9].Type I includes 9 infectious diseases with higher severity but lower incidence (plague, cholera, SARS, human avian influenza, poliomyelitis, anthrax, diphtheria, filariasis and unexplained pneumonia). The fixed threshold detection method (FDM) is applied to detect aberration of type I and the threshold of FDM is a fixed value and usually set as one [10–14]. Type II includes 21 infectious diseases with high incidence but lower severity (Hepatitis A, Hepatitis B, Hepatitis C, measles, epidemic haemorrhagic fever, epidemic encephalitis B, dengue fever, bacillary and amoebic dysentery, typhoid and paratyphoid, epidemic cerebrospinal meningitis, scarlet fever, leptospirosis, malaria, influenza, epidemic mumps, rubella, acute hemorrhagic conjunctivitis, epidemic and endemic typhus, chickenpox, hand foot mouth disease, infectious diarrhoea). The temporal detection method (TDM) [10–12] is applied to more common infectious diseases of Type II [10–12]. Moving percentile method (MPM) [9], the most common TDM, is used to detect aberration of disease occurrence by comparing the reported cases in the current observation period to that of the corresponding historical period at the county level [7]. Once the CIDARS detect a disease aberration by FDM or TDM, the CIDARS would send early alert signals to specific mobile phones by SMS to ensure the accurate and timely dissemination of surveillance information [8]. When local CDC staff receive signals indicating potential outbreaks, they are required to verify the occurrence of these outbreaks via phone interviews or onsite investigations and provide feedback in the CIDARS within 12 h.

In the CIDARS, early alert signals are generated when the number of reported cases exceeds the thresholds [15, 16]. Thus, the selection of a threshold for each infectious disease is crucial for the performance of the CIDARS. In China, CDCs at all level are currently using the thresholds recommended by China CDC, and these recommended thresholds have recognized limitations. Jie Kuang [9] mentioned in his study that the CIDARS generates many false-positive signals, and large differences exist between the outbreak signal counts and the final identified outbreaks, these issues prompted us that national recommended thresholds might be not ideal for all level of CDCs in China. To improve the performance of the CIDARS at outbreak detection, the China CDC has suggested that CDCs at all levels should carry out studies to select proper thresholds according to the epidemic characteristics of local infectious diseases [1, 17, 18]. However, evidence on how to select the proper threshold to detect infectious disease aberrations based on the CIDARS is limited.

In this study, 6 infectious diseases of Type II were selected because of their high morbidity (accounting for 40% of total infectious diseases) and higher frequency of early alarming signals in the CIDARS (accounting for 75% of total early alarming signals) in Shanghai. Our study sought to explore an operational method to select the proper early warning threshold according to the epidemic characteristics of local infectious diseases based on the CIDARS. Findings of this study may therefore help public health practitioners understand the principle ideas behind automated surveillance and provide methodological references for future research and implementation.

## Methods

### Data sources

In this study, we select 6 Type II infectious diseases in the Songjiang district of Shanghai. These 6 infectious diseases include hand foot and mouth diseases (HFMD), mumps, influenza, scarlet fever, chickenpox, and rubella. Data for cases of selected infectious diseases during the period from 2009 to 2015 are extracted from NIDRIS and organized by calendar week (1–52) and by year (2009–2015) in Excel format. Data from 2009 to 2014 are used to establish the model and select the proper early-alert thresholds. Data from 2015 are used as testing data to evaluate the performance of the previously selected early-alert thresholds. Meanwhile, early alerting signals of 6 selected infectious diseases from 2009 to 2014 are extracted from the CIDARS to illustrate the system outputs which was based on the national recommended thresholds.

### Study design

**Fig. 1:**
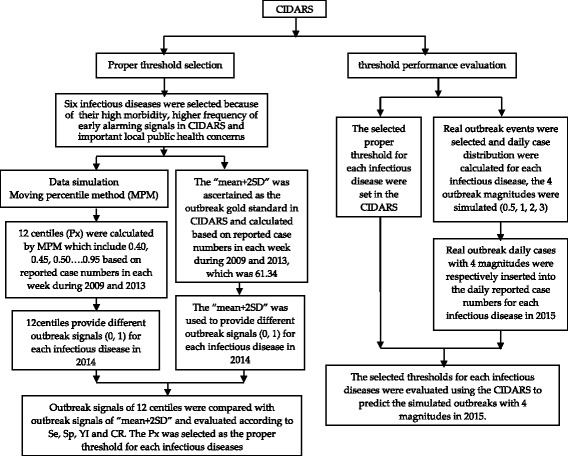
Flowchart of data processing and performance evaluation based on CIDARS

This study has two stages which is depicted in Fig. 1, the stage one is proper threshold selection, and the stage two is threshold performance evaluation.

In stage one, 12 centiles (Px) are calculated by MPM based on case data of the 6 selected infectious diseases during the period from 2009 to 2014. Meanwhile, the outbreak gold standard (mean + 2SD) in the CIDARS that recommended by China CDC is also calculated based on the same case data from 2009 to 2014. Then early alerting signals generated by each of 12 centiles are compared with those signals generated by the “mean + 2SD” as gold standard in terms of sensitivity, specificity and consistency rate respectively to identify the proper thresholds for each infectious diseases.

In stage two, the selected proper thresholds for each infectious diseases are set in the CIDARS, the performance of new selected thresholds are evaluated by inserting simulated outbreak signals into the data from 2015.

### Model building

In the CIDARS, the moving percentile method (MPM) [9] is used in TDM to detect aberrations and determine the proper threshold of P_x_ (P_x_ is a measure indicating the value below x^th^ percentage of observations in a group of observations fall). Aberrations in disease occurrence are detected by comparing the number of cases reported during the current observation period to the number reported during a corresponding historical period at the county level. The number of cases in the current observation period is the sum of the reported cases in the recent week. To maintain the stability of the data, the previous five years are used as the historical period [8], the corresponding historical period includes, for each of the previous five years, the same current week, the two preceding weeks and the two following weeks, this results in 25 weeks of historical data [5 week*5 year]. The percentile of the 25 blocks of historical data is set as the indicator for potential aberration detection. The current observation period and corresponding historical data block period are dynamically moved forward week by week.

We present the data for chickenpox cases reported in the Songjiang District from the NIDRIS to demonstrate the process of model building and predicted value calculation. As described in Table 1, data for chickenpox from 2009 to 2014 were sorted by calendar weeks and by; the predicted value (P_x_) was calculated using a percentile-based moving window ([5 week*5 year], x), where x was defined as one of 12 centiles (0.40, 0.45, 0.50, 0.55, 0.60, 0.65, 0.70, 0.75, 0.80, 0.85, 0.90, 0.95). For instance, in Table 1, the value of P_40_ for week 3 in 2014 was in the 40th percentile of the 25 blocks of historical data (week [1–5], year [2009–2013]), and the predicted value of P_40_ for week 3 was 35. The value of P_90_ for week 5 in 2014 was in the 90th percentile of the 25 blocks of historical data (week [3–7], year [2009–2013]), and the predicted value of P_90_ for week 5 was 43. Then, we compared the number of cases reported during each week in 2014 with the predicted values generated for the corresponding weeks and determined whether an outbreak signal occurred for each of the 12 centiles; if the reported case number exceeded the predicted value of P_x_ during the corresponding week, an outbreak signal was generated. For instance (see Table 1), in 2014, 26 cases were reported in week 8, which exceeded the predicted values of P_40_ (22) and P_45_ (22); therefore, the CIDARS would generate an outbreak signal (an outbreak signal of P_x_ = 1) if the threshold was set as P_40_ or P_45_. However, the number of cases reported in week 8 was smaller than the predicted values of P_90_ (34) and P_95_ (34); thus, the CIDARS would not generate outbreak signals (an outbreak signal of P_x_ = 0) if the threshold was set as P_90_ or P_95_ (see Table 1).

**Table 1:**
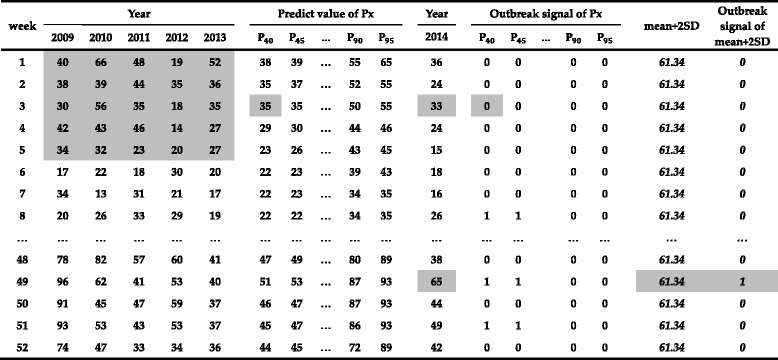
Predicted values and outbreak signals for each percentile value (Px) for chickenpox during 2014 based on the moving percentile method and outbreak signals based on the ‘mean + 2SD’ gold standard in the CIDARS, Songjiang District of Shanghai, China

Px = percentile (moving window [5 week*5 year], x), x = 0.40, 0.45, 0.50,…, 0.85, 0.90, 0.95 (see colored cells, P_40_)

Outbreak signal of Px, 1 = potential outbreak, 0 = not potential outbreak

As depicted in Table 1, for the same infectious disease (chickenpox), we identified the outbreak signals generated by the ‘mean + 2SD’ gold standard method. The ‘mean + 2SD’ was the sum of the average value and 2 times of the standard deviation of 260 weekly reported case numbers from 2009 to 2013, such that the ‘mean + 2SD’ for chickenpox is 61.34. Then, we compared the number of cases reported during each week in 2014 with the ‘mean + 2SD’ gold standard to determine whether an outbreak signal occurred. For instance, in 2014, 65 cases were reported during week 49, which exceeded 61.34, such that the CIDARS would generate an outbreak signal (an outbreak signal of ‘mean + 2SD’ = 1). See Table 1 for additional details.

### Proper early-warning thresholds selection

The proper early warning threshold for each infectious disease is ascertained by comparing the outbreak signals generated by P_x_ with signals generated by the ‘mean + 2SD’ gold standard during the corresponding week. According to the screening theory, a P_x_ value is defined as the proper threshold if the given P_x_ generated outbreak signals are mostly in line with the signals generated by the ‘mean + 2SD’method (see Tables 2 and 3). Se (Sensitivity), Sp (Specificity), YI (Youden’s Index) and CR (Consistency Rate) are used as evaluation indexes.

**Table 2:** Proper threshold selection method and evaluation indexes for the performance of Px

Outbreak signals of Px	Outbreak Gold Standard ‘mean + 2SD’		Total
	1	0	
1	A	B	R1
0	C	D	R2
Total	C1	C2	N

Sensitivity, Se = A/C1; Specificity, Sp = D/C2; Youden’s Index, YI = Se + Sp-1; Consistency Rate, CR = (A + D)/N

Se is defined as the percentage of all positive outbreak signals (1) generated by outbreak gold standard “mean + 2SD” that is also identified by a P_x_, it measures the proportion of positives that are correctly identified. Sp is defined as the percentage of all negative outbreak signals (0) generated the outbreak gold standard “mean + 2SD” that is also justified as negative by a P_x_, it measures the proportion of negatives that are correctly identified. YI is a way of summarizing the performance of a diagnostic test, its value ranges from −1 to 1, and has a zero value when a diagnostic test gives the same proportion of positive results for groups with and without the disease. CR is the ratio of identical signals generated by a P_x_ and using the “mean + 2SD” outbreak gold standard for the total 52 signals, its value ranges from 0 to 1, and larger CR values demonstrate better consistency between the P_x_ and the “mean + 2SD”.

For instance, P_75_ is most in line with signals generated by the ‘mean + 2SD’ gold standard for chickenpox, demonstrating a Se of 100%, a Sp of 90.20%, a YI of 90.20% and a CR of 0.90; therefore, P_75_ is defined as the proper threshold for chickenpox. For additional details, see Table 3.

**Table 3:** The proper threshold Px selection for each infectious disease (e.g., chickenpox) based on the CIDARS, Songjiang District of Shanghai, China

Px	Values based on the ‘mean + 2SD’				Evaluation indexes			
	A	B	C	D	Se (%)	Sp (%)	YI (%)	CR(%)
P_40_	1	22	0	29	100.00	56.86	56.86	57.69
P_45_	1	20	0	31	100.00	60.78	60.78	61.54
P_50_	1	16	0	35	100.00	68.63	68.63	69.23
P_55_	1	13	0	38	100.00	74.51	74.51	75.00
P_60_	1	11	0	40	100.00	78.43	78.43	78.85
P_65_	1	6	0	45	100.00	88.24	88.24	88.46
P_70_	1	6	0	45	100.00	88.24	88.24	88.46
P_75_	1	5	0	46	100.00	90.20	90.20	90.38
P_80_	0	4	1	47	0.00	92.16	−7.84	90.38
P_85_	0	4	1	47	0.00	92.16	−7.84	90.38
P_90_	0	3	1	48	0.00	94.12	−5.88	92.31
P_95_	0	2	1	49	0.00	96.08	−3.92	94.23

### Proper early-warning thresholds validation

**Fig. 2:**
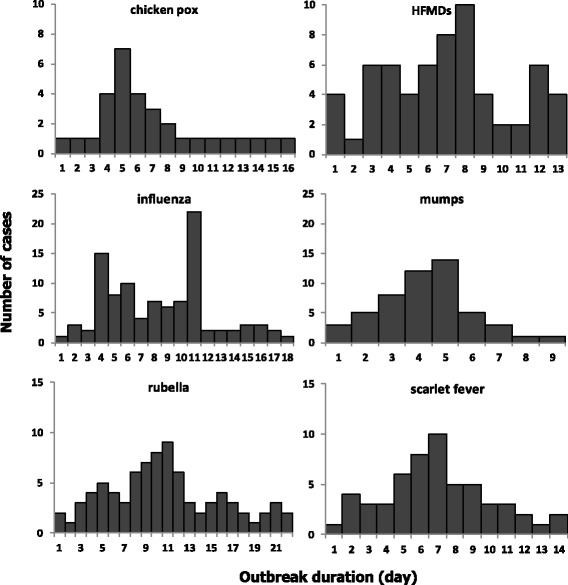
The daily case distribution for 6 outbreaks in the Songjiang District of Shanghai, China

The selected proper early warning thresholds are validated by inserting simulated outbreak signals into the real baseline data in 2015. We select one real outbreak event for each infectious disease in the Songjiang district of Shanghai. All 6 real outbreaks are confirmed through field epidemiological investigation by local CDC staff members. The daily case distribution is calculated from the onset of the outbreak (Fig. 2),which is defined as onset date of the first case. Then, for calculation convenience, we just assume that outbreak curve for each infectious disease scales in a linear fashion across the entire outbreak. We multiples the corresponding baseline case numbers for all days of an outbreak by 0.5, 1.0, 2.0 and 3.0 to simulate 4 outbreak magnitudes. For example, an outbreak magnitude of 0.5 is the product of the baseline case numbers and 0.5.

For each infectious disease, we insert the simulated outbreak on the first day of each month. If the month has a public health emergency (infectious disease outbreaks or epidemics), then we skip the artificial insertion. This gives us 6 types of outbreak signals; each infectious disease has 4 outbreak magnitude test datasets for a total of 24 test datasets. In theory, 288 outbreak signals could be inserted to evaluate the selected proper thresholds. We finally insert 288 outbreak signals.

The early warning performance of the selected proper thresholds for each infectious diseases is based on 3 indicators, sensitivity (the proportion of outbreaks the threshold detected), timeliness (the duration between the first true alarm and the onset of the outbreak), and the false alarm number (alarm signal indicating false outbreak).

### Data analysis

Data analysis is performed using Excel 2013 and SPSS software (version 16.0 for windows). Excel is used to sort the data, and simulate the outbreak detection. SPSS is used to calculate the evaluation indexes (Se, Sp, YI, CR, etc.) and to identify the proper threshold of P_x_ for each infectious disease and to compare the performance of the proper thresholds with the national recommended thresholds.

## Results

### General description

**Fig. 3:**
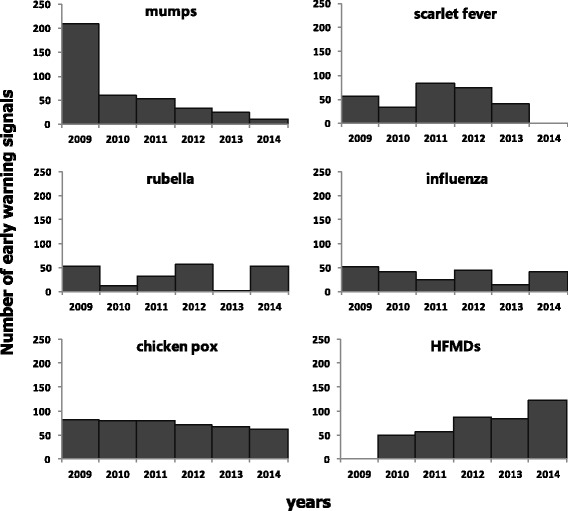
Number of early warning signals for 6 infectious diseases during 2009 and 2014 in the Songjiang District of Shanghai, China

In this study, 6 infectious diseases were selected for study in the Songjiang District of Shanghai. The 6 infectious diseases included HFMD, mumps, influenza, scarlet fever, chickenpox and rubella. According to China CDC’s recommendation for thresholds in the CIDARS, P80 was used for each of 6 selected infectious disease. Early warning signals generated in the CIDARS was described in Fig. 3. The number of early warning signals for mumps decreased obviously during 2009 and 2014, and for HFMD, the number of early warning signal increased slightly each year during 2009 and 2014. However, no obvious increasing or decreasing trends were identified or scarlet fever, rubella, influenza or chickenpox in the number of early warning signals during 2009 and 2014.

### Proper early-alert threshold selection

The proper thresholds (P_x_) for each infectious disease were selected based on local epidemic characteristics, and their corresponding evaluation indexes were listed in Table 4. The proper thresholds for rubella (P_45_), chickenpox (P_75_), influenza (P_75_) and HFMD (P_75_) were lower than threshold (P_80_) recommended by the China CDC. For mumps and scarlet fever, the proper selected threshold P_80_ was identical to the China CDC’s recommendation.

**Table 4:** Proper thresholds identified for the 6 infectious diseases in the Songjiang District of Shanghai, China

Infectious diseases	Proper Early-alert Thresholds	Se (%)	Sp (%)	YI (%)	CR	Thresholds recommended by the China CDC
Chickenpox	P_75_	100.00	90.20	90.20	0.90	P_80_
Mumps	P_80_	100.00	96.15	96.15	0.96	P_80_
Influenza	P_75_	100.00	64.00	64.00	0.65	P_80_
Rubella	P_45_	50.00	60.87	11.87	0.60	P_80_
HFMD	P_75_	100.00	33.33	33.33	0.71	P_80_
Scarlet fever	P_80_	100.00	55.00	55.00	0.65	P_80_

### Proper early-alert threshold performance verification

**Fig. 4:**
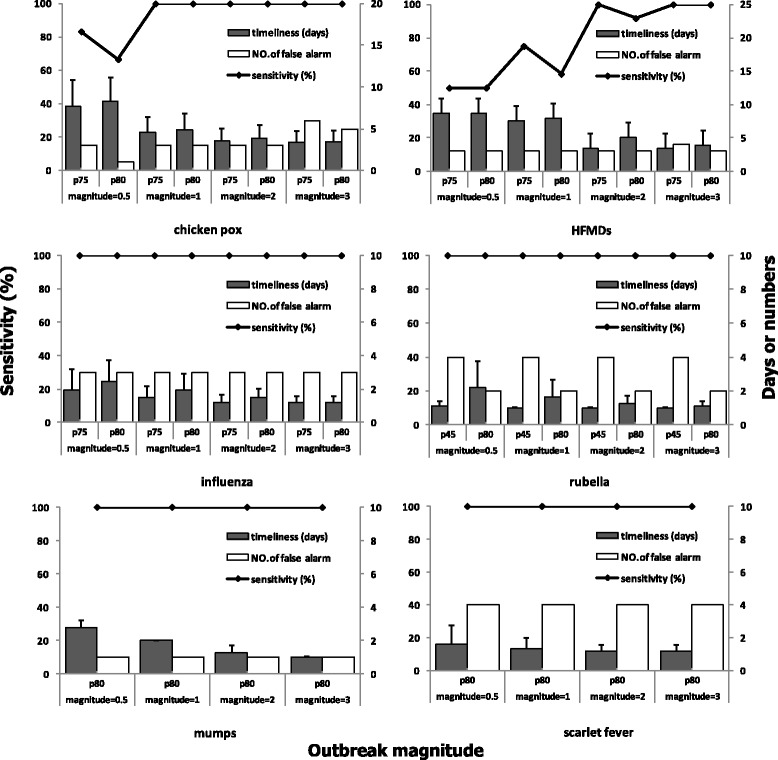
The performance of selected proper thresholds for 6 infectious diseases and thresholds recommended by the China CDC

As described in Fig. 4, for these 6 infectious diseases, almost all simulated outbreaks were detected both by selected proper thresholds (P_75_ for chickenpox, influenza and HFMD, P_80_ for mumps and scarlet fever, P_45_ for rubella) and the national recommended thresholds (P_80_ for all 6 infectious disease) except for chickenpox and HFMDS. For chickenpox at 0.5 magnitude and HFMD at 1 and 2 magnitudes, the sensitivity of the selected proper threshold was higher than the national recommended threshold. In terms of timeliness, the selected proper thresholds required shorter time periods to detect the outbreaks compared with national recommended thresholds, to take chickenpox as an example, the detection days were 2 days, 1 day, 0.5 day and 0.5 day earlier for magnitude 0.5, 1, 2 and 3 respectively. Meanwhile, the larger the outbreak magnitude, the shorter the time needed to detect outbreaks. However, the selected proper thresholds were prone to generate more false alarms in comparison with the national recommended thresholds. (Fig. 4).

## Discussion

Jie Kuang [9] previously reported that the moving percentile method (MPM) was a useful algorithm for outbreak detection. The CIDARS therefore adopted the MPM to detect infectious disease aberrations and determine the proper threshold of P_x_ for common infectious diseases. In this study, based on the characteristics of local infectious diseases, we selected the proper P_x_ threshold for 6 infectious diseases that were major public health problems in the Songjiang district. The performance of thresholds demonstrated that, the selected proper thresholds could detect almost all simulated outbreaks within a shorter time period than the thresholds recommended by the China CDC. This study indicates that it is crucial to select the proper threshold to detect infectious disease aberrations based on the characteristics of local diseases in the CIDARS, which can improve the performance of outbreak detection.

One major challenge in outbreak detection evaluation is obtaining a sufficient number of outbreak data with which to measure sensitivity and timeliness of a given period [19, 20]. For this reason, injecting simulated outbreaks into real surveillance data is a feasible approach [8–10]. In this study, we employed real infectious disease outbreaks that previously occurred in our district as the simulated outbreaks; this approach helped diminish the risk that the simulated data might completely unlike real outbreaks and helped alleviate concerns that outbreaks in the real surveillance data may interfere with the performance evaluation.

Many determinants affect the performance of outbreak detection in automated surveillance, and understanding how these factors affect the detection performance can assist in the improvement of outbreak detection in the CIDARS. Previous studies [20, 21] have reported that system factors (representativeness, outbreak detection algorithms and algorithm specifics), outbreak characteristics (outbreak size, shape of the outbreak signal and time of outbreak) are determinants that can affect outbreak detection. In this study, the outbreak magnitude was considered when injecting simulated outbreak signals to study the performance of selected proper thresholds. Study results demonstrated that larger outbreak magnitude could be detected more quickly and with higher sensitivity. This epidemic feature of infectious diseases should thus be considered for similar studies.

Previously, Xiao-Li Wang et al. [22, 23] reported that the morbidity and mortality associated with infectious diseases and the CDCs’ emergency response ability should be taken into consideration during the proper threshold selection process based on data from the CIDARS. Based on the results of our study, we suggest that proper threshold selection should take the epidemic feature as well as the local infectious disease characteristics into consideration. A lower threshold may be preferable if the evaluated infectious disease, such as influenza, is associated with a tremendous threat and has reliable treatment and control measures, as a lower threshold could improve sensitivity and identify more potential outbreaks. However, it may be wiser to select a relatively higher threshold when the infectious disease has mild effects but the cost of investigation and control is high because this could reduce the generation of false warning signals and improve the efficiency of infectious disease control and prevention.

There are some limitations to this study. First, we only selected 6 infectious diseases with higher associated morbidity and more early-alert signals to evaluate in this study. However, infectious diseases with lower associated morbidity may differ from these key infectious diseases. Second, as only using 1 year test datasets, we were only able to insert a limited number of simulated outbreaks. This may in turn have affected the stability of our evaluation to some extent. Third, we only employed the outbreak magnitude to evaluate the influence of outbreak detection performance, however, the other epidemic features include the incubation period and baseline counts may also affect the performance of outbreak detection. The incorporation of some improvements should be considered in further studies. Fourth, the unmeasured factors that affect outbreaks are changing over time, and the use of a historical threshold to test current and future values may fail to address the effect of confounding factors. For this reason, bidirectional sampling of training data would allow a middle year to be tested with control for confounders. Furthermore, investigating more infectious diseases with different epidemic characteristics may improve the generalizability of our study findings. Pre-diagnosis data, such as data from hospitals, media reports, school absenteeism and drugstores that may identify cases could be considered for integration with current notifiable infectious disease surveillance data, thereby potentially improving the sensitivity and timeliness of the current CIDARS.

## Conclusions

It is crucial to select the proper early-warning thresholds for the detection of infectious disease aberrations based on the characteristics and epidemic feature of local infectious diseases in the CIDARS, which could improve the performance of outbreak detection.

## Abbreviations

CDC: Centre for Disease Control and Prevention
CIDARS: China Infectious Disease Automated-alert and Response System
FDM: Fixed Threshold Detection Method
HFMD: Hand Foot Mouth Diseases
NIDRIS: Nationwide Notifiable Infectious Diseases Reporting Information System
TDM: Temporal Detection Method
